# Life’s Essential 8 is associated with atherosclerotic cardiovascular disease but not venous thromboembolism in men: a prospective cohort study

**DOI:** 10.1080/07853890.2023.2233894

**Published:** 2023-07-17

**Authors:** Nzechukwu M. Isiozor, Jari A. Laukkanen, Ari Voutilainen, Isabela M. Bensenor, Setor K. Kunutsor

**Affiliations:** a Institute of Clinical Medicine, University of Eastern Finland, Kuopio, Finland; b Institute of Public Health and Clinical Nutrition, University of Eastern Finland, Kuopio, Finland; c Department of Internal Medicine, Central Finland Health Care District, Jyvaskyla, Finland; d School of Medicine, University of São Paulo, São Paulo, Brazil; e Diabetes Research Centre, University of Leicester, Leicester General Hospital, Leicester, UK

**Keywords:** Cardiovascular disease, venous thromboembolism, atherosclerotic cardiovascular disease, life’s essential 8, cardiovascular health metrics, risk factors, cohort study

## Abstract

**Background:** Atherosclerotic cardiovascular disease (ASCVD) shares several risk factors with venous thromboembolism (VTE). The American Heart Association’s Life’s Simple 7 (LS7), which included seven health and behavioural factors for CVD prevention, has recently been upgraded to Life’s Essential 8 (LE8) score. We aimed to examine the prospective association between LE8 and the risks of ASCVD and VTE in Finland.

**Methods:** We utilized data based on 1899 men aged 42–61 years in the Kuopio Ischaemic Heart Disease (KIHD) prospective study. The LE8 score was generated from baselines measures of four health behaviours (physical activity, diet, nicotine exposure and sleep health) and four health factors (BMI, blood lipids, blood glucose and blood pressure). Each factor was scored from 0 to 100 and summed into a composite score. Participants were classified into quartiles (Q) based on the total LE8 score – Q1, ≤ 420; Q2, >420 to 485; Q3, >485 to 550; Q4, >550. Multivariable Cox regression models were utilized to determine the hazard ratios (HRs) along with the 95% confidence intervals (CI) for ASCVD and VTE.

**Results:** After median follow-up durations of 24 and 25 years, 889 ASCVD and 127 VTE events were recorded, respectively. The risk of ASCVD was found to be 58% lower in men belonging to the highest LE8 quartile compared to those in the lowest quartile (HR:0.42; 95%CI: 0.34–0.51). There was no significant evidence of an association between LE8 and VTE risk (Q4 vs Q1, HR:1.02; 95%CI: 0.60–1.74).

**Conclusion:** The risk of ASCVD was significantly lower in middle-aged and older Finnish men who had a high LE8 score, but there was no significant association with VTE. Further large-scale prospective studies conducted in women and other population groups are necessary to confirm these findings.

## Summary

Men who had total Life’s Essential 8 (LE8) score more than 550 had lower risk of atherosclerotic cardiovascular disease (ASCVD) compared with those with LE8 score ≤ 420.

Increasing LE8 score by 50 can lower the risk of ASCVD, but not venous thromboembolism (VTE).

The result supports continuous improvement in combined healthy behaviours and factors in LE8 composite score may lower future risk of heart disease or stroke.

## Introduction

Cardiovascular disease (CVD) remains the leading cause of death worldwide, with atherosclerotic cardiovascular disease (ASCVD) being the most common subtype. ASCVD manifests mostly as coronary heart disease (CHD) and stroke, which are major contributors to disability [1,2]. In Finland, CVD accounts for 33% of mortality, with 1 in every 6 deaths caused by CHD (3). Venous thromboembolism (VTE) manifests generally as deep vein thrombosis and pulmonary embolism and is a leading cause of lost disability-adjusted life-years worldwide [3,4]. Although the mechanisms linking the ASCVD and VTE conditions are complex and likely to involve inflammation, coagulation and endothelial dysfunction [3,5], evidence suggests ASCVD and VTE share common underlying pathology and risk factors such as older age, obesity and smoking [6,7]. Given the overlapping nature of these disease conditions, prevention strategies and interventions directed towards the modifiable risk factors are achievable goals. These could reduce the risk of both ASCVD and VTE and their associated burden in general population.

In 2010, the American Heart Association (AHA) developed cardiovascular health metrics, termed Life’s Simple 7 (LS7) which included seven health and behavioural factors for CVD prevention and cardiovascular health improvement [8]. Optimal levels of LS7 have been consistently shown to be associated with a lower risk of adverse cardiovascular outcomes in various populations [9–12]. Quite recently, this metrics was upgraded to Life’s Essential 8 (LE8) with a new factor included to the earlier LS7 factors and the scoring method modified [13]. The new LE8 factors include physical activity (PA), body mass index (BMI) and blood pressure (BP), with updated factors as diet, nicotine exposure, blood lipids, blood glucose and the new factor is sleep. The quantification of each factor ranges from point 0 to 100 (Supplementary Table 1).

Previous studies have reported inverse associations between optimal LS7 score and risk of VTE [14,15] and ASCVD [16]. However, since the launch of LE8 in 2022, no study has evaluated its prospective association with VTE risk; furthermore, only one study in China has reported the association of LE8 with ASCVD [17]. Using data based on a northern European population with a high burden of CVD, we aimed to examine the prospective relation between LE8 and the risk of ASCVD and VTE.

## Methods

The study was reported following the guidelines provided by Strengthening the Reporting of Observational studies in Epidemiology (STROBE), which is a recommended protocol for reporting observational studies in epidemiology (STROBE checklist attached).

This study is based on the Kuopio Ischaemic Heart Disease (KIHD) risk factor study, a general population prospective cohort study comprising of 2682 eastern Finnish men who were 42 – 61 years at baseline examination (1984–1989). The KIHD was designed to investigate various risk factors for ASCVD and other chronic diseases [18]. Details of this have been described earlier [19]. The present analysis is based on 1899 men with complete information on LE8, relevant covariates and outcomes, following exclusion of those with a history of CHD (677), stroke (38) or claudication (64). The Research Ethics Committee of the University of Eastern Finland approved the study (reference number: 143/97), and the protocol adhered to the ethical guidelines of the Helsinki Declaration. All participants gave informed consent.

Self-administered questionnaires were sent to participants before their visits to the study centre. Interviews and clinical examinations were carried out at the study centre. A trained research nurse performed the interviews. Details of the assessment for BP, BMI, nutritional status, nicotine exposure, smoking status, alcohol intake, PA, prevalent medical conditions and socioeconomic status (SES) have been previously described [20]. Diet was assessed using a four-day food record diary, during three weekdays and one weekend day using household measures. A visual aid was utilized in the form of a picture book containing images of familiar foods and dishes to assist participants in estimating portion sizes. Participants were given the option to select portion sizes from three to five commonly used measurements or describe the portion size relative to those in the picture book. To enhance accuracy, instructions were provided, and food records were reviewed by a nutritionist in conjunction with the participants. We analysed food consumption and nutrient intake using The Micro NUTRICA version 2.5 software (Social Insurance Institution, Finland), that uses Finnish and international data on the nutrient compositions of foods [21]. Conditioning PA (≥ 3 metabolic equivalents (METs) was assessed with a seven-day PA diary. The participants were queried about the duration and intensity level (categorized as 0 for recreational, 1 for conditioning, 2 for brisk conditioning and 3 for competitive, strenuous exercise) of all activities they engaged in during the prior week. Additionally, they were asked to report the distance covered through walking, jogging, skiing, bicycling and swimming during the same time period [22]. To evaluate nicotine exposure, a set of questions based on self-reporting was utilized. These questions include: (1) Whether the individual has smoked before (answered with yes or no), (2) The current smoking status of the individual (answered with no, irregularly or regularly), (3) The last time the individual smoked and (4) The frequency of exposure to a smoky environment.

In this study, the SES of adults was measured using self-reported questionnaires that considered multiple factors, including income, occupational prestige, education, material standard of living and housing conditions. Income was categorized into quintiles based on the past 12 months. Participants’ occupational status was classified into three groups: white collar (including professional and managerial staff and low-paid clerical workers), blue collar (including manual labourers in construction, mining, manufacturing or forestry) and farmer (including those who primarily worked in the agricultural sector). Education was divided into four categories: less than elementary education, completion of elementary education, completion of middle school or part of middle school and completion of high school or above. Standard of living was assessed using a self-reported material possession index based on ownership of 12 items (such as colour TV, video tape recorder, freezer, dish washer, car, motorcycle, telephone, summer cottage, house trailer, motor boat, sailing boat and ski mobile). The combined SES scale had a range of 0 to 25, with higher values indicating lower SES [19]. The duration of sleep was evaluated by asking the participants to report how many hours they typically sleep each night. The Nordic Alcohol Consumption Inventory was utilized to assess the consumption of alcohol [23].

Weight and height measured at baseline examination were used to calculate the BMI (weight in kilograms divided by height in metres squared (kg/m^2^)). Blood pressure was measured before 10 a.m. in supine, sitting and standing positions using a random-zero mercury sphygmomanometer (Hawskley, UK). The average of the BP values was used for both systolic and diastolic BP. Blood samples were collected from participants between 8 a.m. and 10 a.m. in the morning, after 12 h of fasting and smoking and 72 h abstinence from alcohol consumption. After 30 min of rest in a supine position, blood sample was drawn from the antecubital vein with Terumo Venoject VT-100PZ vacuum (Terumo Corp., Tokyo, Japan), without the use of tourniquet. Glucose dehydrogenase method was used to measure blood glucose. The cholesterol contents of serum lipoprotein fractions and triglycerides were measured enzymatically (CHOD-PAP, Boehringer, Mannheim, Germany). Using ultracentrifugation and precipitation, serum high-density lipoprotein cholesterol and its subfractions were separated from fresh serum samples.

### Life’s Essential 8

The LE8 scores were measured according to the recently updated AHA’s CVH metrics [13] (Supplementary Table 1), consisting of eight factors, i.e. four health behaviours (diet, PA, nicotine exposure and sleep health) and four health factors (BMI, blood lipids, blood glucose and BP). Scores for each factor were assigned using the AHA’s newly developed scale ranging from 0 to 100. Thus, the overall LE8 score ranged from 0 to 800. The highest and lowest points for each LE8 factor are described below:

- Diet: The Mediterranean Eating Pattern for Americans (MEPA) score was used (Supplementary Table 2). The lowest MEPA score range of 0–3 was assigned the least point of 0, while the highest MEPA score of 15–16 has highest point of 100.
- Physical activity (PA): Moderate or vigorous PA of ≥150 min/week = 100 points; no PA/week = 0 point
- Nicotine exposure (including cigarettes, inhaled nicotine-delivery system and second-hand exposure): never smoked and no second-hand exposure = 100points; current smokers = 0 point
- Sleep health (average sleep/night): 7 to <9 h= 100 points; < 4 h = 0 points
- Body mass index (kg/m^2^): <25 = 100 points; ≥40.0 = 0 point
- Blood lipids (non-high density lipoprotein cholesterol, mg/dL): <130 = 100 points; ≥220 = 0 point. However, for drug-treated level, 20 points was subtracted.
- Blood glucose (fasting, mg/dL or HbA1c, %): No history of diabetes and FBG <100 (or HbA1c <5.7) = 100 points; diabetes with HbA1c ≥10 = 0 point.
- Blood pressure (systolic/diastolic, mmHg): <120/<80 (optimal) = 100 points; systolic of ≥160 or diastolic of ≥100 = 0 point. If values are of treated level, 20points was deducted.

To determine the LE8 score, each factor was assigned a point value ranging from 0 (worst) to 100 (best). The cumulative scores for all eight factors were then calculated, resulting in the overall LE8 score.

### Ascertainment of follow-up events

The primary outcomes were ASCVD and VTE. All first incident cases of ASCVD (CHD or stroke) and incident VTE events were included, from study entry until end of 2018. The Finnish part of Monitoring of Trends and Determinants in Cardiovascular Diseases (FINMONICA) stroke register was used to ascertain the incident strokes between 1984 and 1992. Other events, including CHD, VTE and incident strokes (from 1993), were identified by computer linkages to National Hospital Discharge Registry Data maintained by the Finnish Institute for Health and Welfare. The diagnoses for VTE required positive imaging tests. All medical documents for each potential VTE case were cross-checked in detail, and each event was validated by two physicians who were blinded to the exposures. The International Classification of Diseases (ICD)-10 codes (I26, I80 and I82) were used to code and classify each VTE case; ICD-10 codes I20–I25 for CHD and ICD-9 (codes 430–438) and ICD-10 (codes I60–I68 and G45–G46) for stroke events. The diagnostic classification of CHD was based on symptoms, electrocardiographic findings, cardiac enzyme elevations and outcomes [11,24].

### Statistical analysis

Descriptive statistics were utilized to provide an overview of the baseline characteristics of the participants. Continuous variables were presented as either mean ± standard deviation or median (interquartile range), while categorical variables were expressed as frequencies (percentage). The differences in means, median or percentages were evaluated using the *t*-test, Mann–Whitney U test or *chi*-squared test, respectively. All the participants were followed up from baseline until the date of first events (ASCVD and VTE), death or end of the follow-up, whichever came first.

The LE8 scores were divided into quartiles based on the distribution in the population: quartile 1 (Q1) with scores ≤420, quartile 2 (Q2) with scores >420–485, quartile 3 (Q3) with scores >485–550 and quartile 4 (Q4) with scores >550. The hazard ratios (HRs) and 95% confidence intervals (CIs) for ASCVD and VTE were estimated using multivariable Cox regression models, after confirming no major departure from the proportionality assumptions using Schoenfeld residuals [25]. Participants within the first quartile LE8 score were treated as the reference group to estimate the protective effect of the LE8. The LE8 score was also modelled as a continuous variable (per 50-unit increment). Based on the two domains of CVH metrics, i.e. health behaviours and health factors, LE8 health behaviour and LE8 health factor scores were created. An LE8 score excluding sleep health was also generated. Further analysis was done to evaluate the effect of each LE8 factor (as recently updated) has on outcomes. The shape of the relationship between LE8 (as continuous variable) and the risk of outcomes was explored using restricted cubic splines with knots at the 5th, 35th, 65th and 95th of LE8 distribution in a multivariable adjusted model.

To estimate the HRs, two adjusted models were utilized. The first model was adjusted solely for age, while the second model incorporated additional adjustments for alcohol consumption, SES and family history of CHD. All participants with history of CVD were excluded in the sensitivity analyses. All statistical analyses were conducted using IBM SPSS Statistics 27 and R software. A two-sided *p* value < .05 was considered statistically significant.

## Results

**Figure 1.**
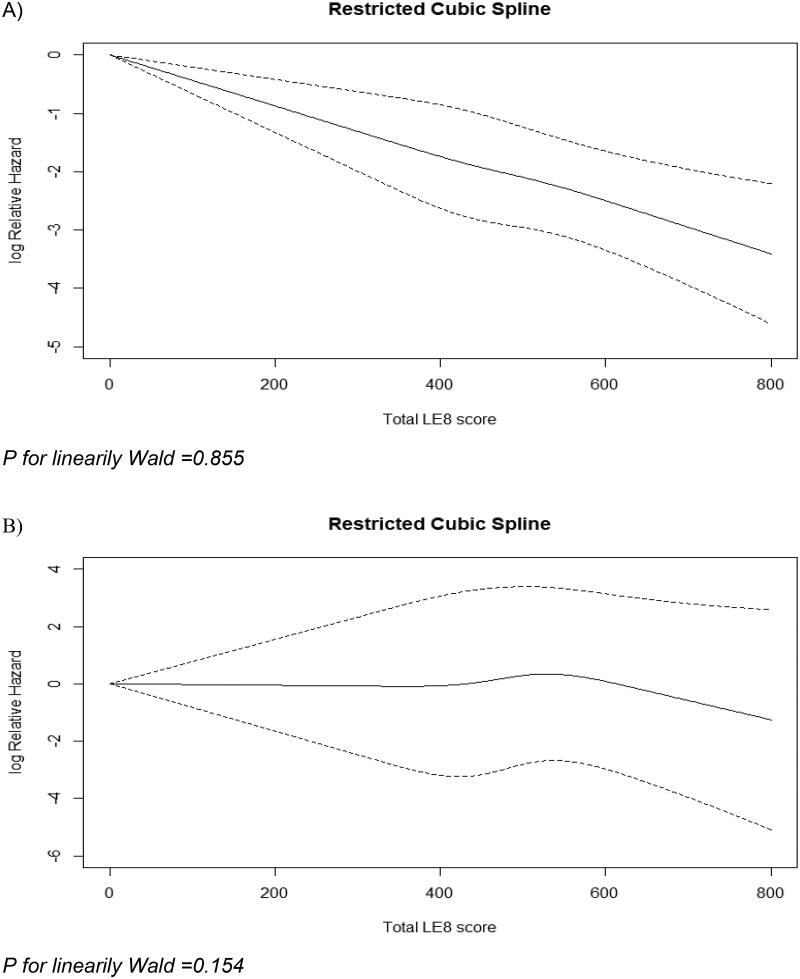
Restricted cubic curve for atherosclerotic cardiovascular disease (A) and venous thromboembolism (B) against life’s Essential (LE8) score. *p* for linearity Wald = .855. *p* for linearity Wald =.154.

The baseline characteristics of the 1899 included participants are shown in Table 1. A total of 889 ASCVD and 127 VTE events were recorded after median follow-up durations of 24 and 25 years, respectively. The study participants had a mean age of 52 years, with the individuals diagnosed with ASCVD and VTE being comparatively older with a mean age of 53 years for ASCVD and 54 years for VTE. Among all participants, 30% were smokers (active/past) and 47% reported a family history of CHD. For the LE8 factors among all the participants, diet had the least mean score of 29.5, whereas blood glucose had the highest mean score of 95.8. Whereas there were significant differences in means across all the four LE8 health factor components’ scores for ASCVD status, only PA, among the health behaviour components, showed a statistically significant difference with higher means in people with no ASCVD. However, the mean difference for nicotine exposure was significantly higher in people with VTE compared to others. The overall LE8 score ranged from 185 to 750, with a mean score of 482.6. This average is 60% of the maximum LE8 score possible (i.e. 482.6 out of 800). A restricted cubic spline showed that the risk of ASCVD, but not VTE, decreased continuously with increasing LE8 scores across the range of 0–800 (Figure 1). Most participants with ASCVD (273) and VTE (42) exhibited scores falling within the first quartile (Q1, ≤ 420) and third quartile (Q3, >485 to 550) of the LE8 distribution, respectively.

**Table 1.** Baseline characteristics of the KIHD study cohort.

Characteristics	All participants (*N* = 1899)	No ASCVD (*N* = 1010)	ASCVD (*N* = 889)	*p* Value	No VTE (*N* = 1772)	VTE (*N* = 127)	*p* Value
Age (years)	52.3 ± 5.3	51.6 ± 5.6	53.1 ± 4.9	<.01	52.2 ± 5.4	53.7 ± 4.5	<.01
Socioeconomic status	11.7 ± 5.1	11.4 ± 5.2	12.0 ± 5.1	<.01	11.7 ± 5.1	11.8 ± 5.3	.75
Alcohol/week (g)	32.0	33.6	30.1	.06	32.0	30.1	.39
	(6.4–97.7)	(7.0–97.7)	(6.1–86.5)		(6.4–92.3)	(5.5–81.3)	
Systolic blood pressure (mmHg)	134.2 ± 16.5	133.1 ± 16.5	135.4 ± 16.3	<.01	134.3 ± 16.6	132.8 ± 14.2	.31
Diastolic blood pressure (mmHg)	89.1 ± 10.5	88.2 ± 10.6	90.1 ± 10.2	<.01	89.1 ± 10.5	88.9 ± 9.6	.88
Smoker	571 (30.1)	297 (29.4)	274 (30.8)	.50	552 (31.2)	19 (15.0)	<.01
History of diabetes	90 (4.7)	36 (3.6)	54 (6.1)	.01	87 (4.9)	3 (2.4)	.19
History of cancer	34 (1.8)	24 (2.4)	10 (1.1)	.04	30 (1.7)	4 (3.1)	.23
Family history of CHD	896 (47.2)	460 (45.5)	436 (49.0)	.13	854 (48.2)	42 (33.1)	<.01
Body mass index (kg/m^2^)	26.7 ± 3.5	26.5 ± 3.4	27.0 ± 3.6	<.01	26.7 ± 3.5	26.8 ± 3.5	.63
Life’s Essential 8 (LE8) factor scores (out of 100 points)							
Diet score	29.5 ± 11.0	29.4 ± 11.1	29.5 ± 10.8	.86	29.5 ± 11.1	29.1 ± 9.8	.71
Physical activity score	56.8 ± 44.0	59.2 ± 43.3	54.1 ± 44.6	.01	56.8 ± 43.9	55.84 ± 44.3	.79
Nicotine exposure score	55.1 ± 41.5	55.6 ± 41.5	54.6 ± 41.5	.61	54.2 ± 41.7	68.2 ± 36.2	<.01
Sleep health score	90.8 ± 18.8	90.7 ± 18.7	90.8 ± 18.9	.94	90.8 ± 18.7	90.1 ± 19.8	.67
Body mass index score	73.5 ± 24.1	75.1 ± 23.6	71.8 ± 24.5	<.01	73.6 ± 24.2	72.9 ± 22.6	.75
Blood lipid score	42.8 ± 29.2	46.1 ± 29.2	39.0 ± 28.7	<.01	42.6 ± 29.3	44.9 ± 27.9	.40
Blood glucose score	95.8 ± 15.2	96.7 ± 13.4	94.7 ± 17.0	.01	95.6 ± 15.5	97.6 ± 10.9	.15
Blood pressure score	38.4 ± 29.9	41.4 ± 30.5	35.0 ± 28.9	<.01	38.5 ± 30.0	36.9 ± 28.7	.55
							
LE8 score	482.6 ± 92.3	494.2 ± 92.0	469.4 ± 90.9	<.01	481.7 ± 92.9	495.5 ± 82.5	.10
LE8 (in quartiles, Q)				<.01			.07
Q1, ≤ 420	494 (26.0)	221 (21.9)	273 (30.7)		468 (26.4)	26 (20.5)	
Q2, >420 to 485	495 (26.1)	257 (25.4)	238 (26.8)		467 (26.4)	28 (22.0)	
Q3, >485 to 550	457 (24.1)	245 (24.3)	212 (23.8)		415 (23.4)	42 (33.1)	
Q4, >550	453 (23.9)	287 (28.4)	166 (18.7)		422 (23.8)	31 (24.4)	

*Notes:* ASCVD: atherosclerotic cardiovascular disease; CHD: coronary heart disease; KIHD: Kuopio Ischaemic Heart Disease; LE8: Life’s Essential 8; VTE: venous thromboembolism. *p* Values for t-tests, Mann*–*Whitney U test and chi-squared tests. Data presented as mean **±** standard deviation **(**SD), median (interquartile range) or no. (%) values. Socioeconomic status (SES) was defined as a combined measure of income, education, occupational prestige, material standard of living and housing conditions. The scale ranges from 0 to 25, with 0 indicating the highest and 25 the lowest.

**Figure 2.**
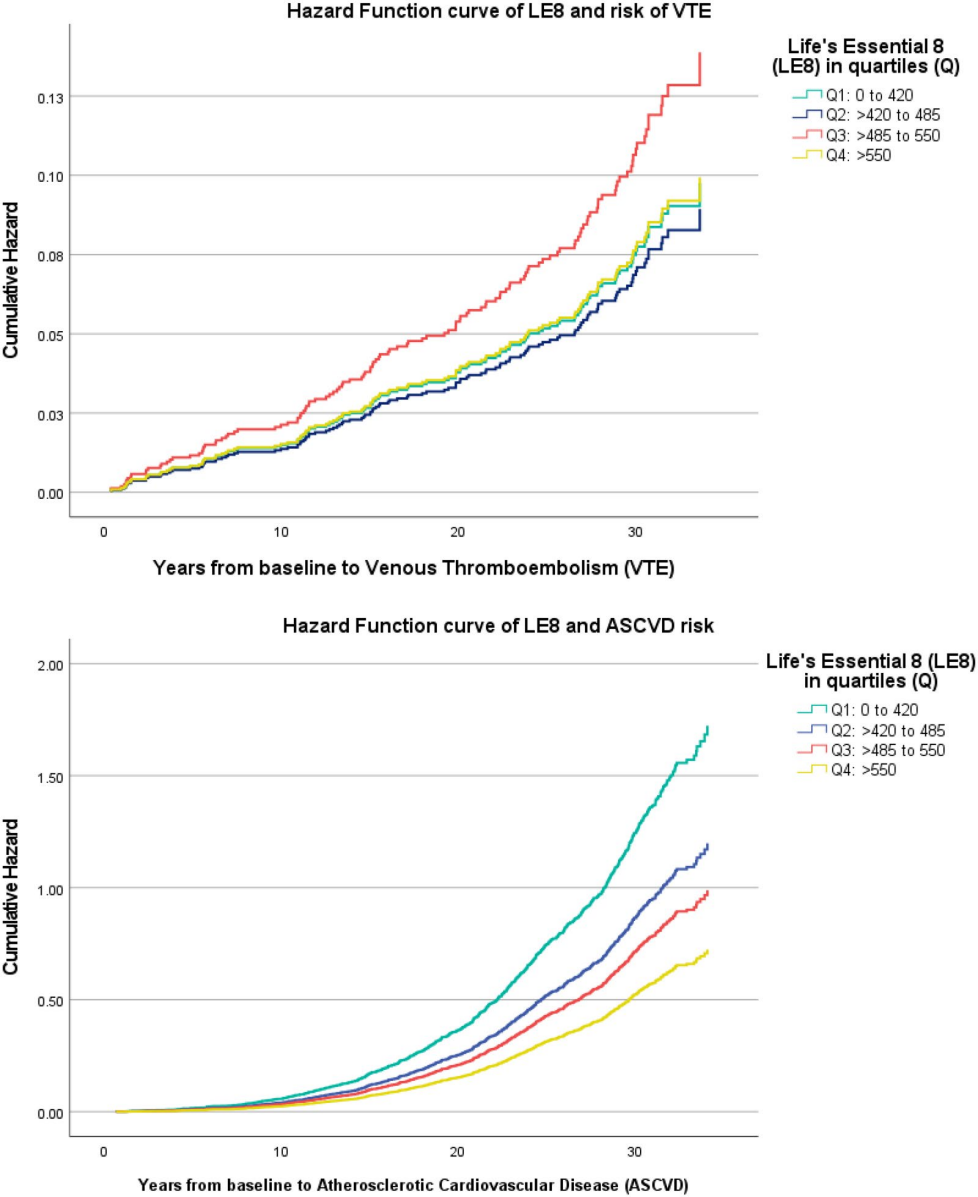
The hazard function curves of LE8 and ASCVD and VTE risks. *p* Value (log rank): <.001 for ASCVD and 0.245 for VTE.

The HRs (95% CIs) for the associations between LE8 and risk of ASCVD and VTE are displayed in Table 2. Higher LE8 quartiles were significantly associated with lower risk of ASCVD, but not for VTE risk. Following adjustments for age, alcohol consumption, SES and family history of CHD, men within the top LE8 quartile (Q4) had 58% lower risk of ASCVD compared to those in the bottom quartile (Q1). The hazard function curves of LE8 and ASCVD and VTE risks are shown in Figure 2. Furthermore, a 50-unit increase in LE8 score was associated with a 17% lower risk of ASCVD. However, we did not observe statistically significant associations for the risk of VTE (HR (95% CI) for Q4 vs. Q1: 1.01 (0.59–1.72) and for 50-unit LE8 score increment: 1.02 (0.92–1.13)). In sensitivity analysis, the findings were similar on exclusion of all participants with a history of CVD (Supplementary Table 3). Further evaluations using the LE8 behaviour and health scores demonstrated similar patterns such that 50-unit increase in LE8 behaviour and health scores were associated with 11% and 25% lower risk of ASCVD, respectively. The pattern remained for ASCVD risk upon exclusion of sleep health, the newly added CVH factor (HR (95% CI): 50-unit LE8 score increment − 0.82 (0.79–0.85)). No significant association was found between the LE8 health behaviour score, LE8 health factor score, LE8 score (excluding sleep health) and the risk of VTE (Table 2).

**Table 2.** LE8 association with ASCVD and VTE in the KIHD study.

	ASCVD			VTE		
	n/N	Model 1	Model 2	n/N	Model 1	Model 2
	889/1899	HR(95%CI); *p* value	HR(95%CI); *p* value	127/1899	HR(95%CI); *p* value	HR(95%CI); *p* value
LE8 (in quartiles, Q)						
Q1	273/494	1(Ref.)	1(Ref.)	26/494	1(Ref.)	1(Ref.)
Q2	238/495	0.67 (0.57–0.80); <.01	0.69 (0.58–0.83); <.01	28/495	0.91 (0.54–1.56); .74	0.94 (0.55-1.60); .81
Q3	212/457	0.55 (0.46–0.66); <.01	0.57 (0.48–0.69); <.01	42/457	1.42 (0.87–2.32); .16	1.46 (0.89–2.39); .13
Q4	166/453	0.39 (0.32–0.47); <.01	0.42 (0.34–0.51); <.01	31/453	1.00 (0.60–1.69); .99	1.01 (0.59–1.72); .98
Total LE8 score (per 50-unit increase)	889/1899	0.82 (0.79–0.85); <.01	0.83 (0.79–0.86); <.01	127/1899	1.02 (0.93–1.12); .70	1.02 (0.92–1.13); .69
LE8 health behaviour score (per 50-unit increase)	889/1899	0.87 (0.83–0.91); <.01	0.89 (0.85–0.93); <.01	127/1899	1.05 (0.92–1.20); .45	1.06 (0.93–1.21); .42
LE8 health factor score (per 50-unit increase)	889/1899	0.73 (0.69–0.78); <.01	0.75 (0.70–0.79); <.01	127/1899	0.98 (0.84–1.14); .78	0.98 (0.84–1.14); .75
LE8 score without sleep health (per 50-unit increase)	889/1899	0.81 (0.78–0.84); <.01	0.82 (0.79–0.85); <.01	127/1899	1.03 (0.93–1.14); .55	1.03 (0.93–1.14); .54
LE8 factors (per 10-unit increase)	889/1899			127/1899		
Diet score		0.99 (0.93–1.05);.66	1.00 (0.94–1.06);.96		0.95 (0.81–1.12); .53	0.94 (0.80–1.11); .45
Physical activity score		0.98 (0.97–1.00);.01	0.99 (0.97–1.00);.04		0.99 (0.95–1.03); .70	1.00 (0.96–1.04); .80
Nicotine exposure score		0.95 (0.94–0.97); <.01	0.96 (0.94–0.98); <.01		1.06 (1.01–1.11); .02	1.06 (1.01–1.11); .02
Sleep health score		0.97 (0.93–1.00); .05	0.97 (0.93–1.00); .07		0.96 (0.87–1.05); .32	0.96 (0.87–1.05); .33
Body mass index score		0.93 (0.90–0.95); <.01	0.93 (0.91–0.96); <.01		0.97 (0.90–1.05); .47	0.97 (0.90–1.05); .49
Blood lipid score		0.94 (0.92–0.96); <.01	0.94 (0.92–0.97); <.01		1.02 (0.97–1.08); .45	1.02 (0.96–1.08); .47
Blood glucose score		0.87 (0.83–0.94); <.01	0.87 (0.83–0.90); <.01		1.07 (0.91–1.25); .41	1.06 (0.91–1.24); .46
Blood pressure score		0.92 (0.90–0.94); <.01	0.93 (0.90–0.95); <.01		0.97 (0.91–1.03); .27	0.97 (0.91–1.03); .26

*Notes:* ASCVD: atherosclerotic cardiovascular disease; CI: confidence interval; HR: hazard ratio; KIHD: Kuopio Ischaemic Heart Disease; LE8: Life’s Essential 8; n/N: number of events/total; VTE: venous thromboembolism. Model 1: adjusted for age. Model 2: Model 1 plus alcohol consumption; socioeconomic status and family history of coronary heart disease. LE8 scores: quartile 1, ≤ 420; quartile 2, >420 to 485; quartile 3, >485 to 550; quartile 4, >550.

The respective associations between the individual components of LE8 and the risks of ASCVD and VTE are presented in Table 2. After full adjustments (model 2), we observed that a 10-unit increase in physical activity score and nicotine exposure score (for the LE8 behaviour factors) were significantly associated with a 1% and 4% lower risk of ASCVD, respectively. Conversely, the risk of VTE increased by 6% with a 10-unit increase in nicotine exposure score. In contrast to the non-significant association with the risk of VTE, a 10-unit increase in all LE8 health factor component scores showed a significantly lower association with the risk of ASCVD. Specifically, a 10-unit increase in BMI, blood lipid, blood glucose and BP scores were associated with a 7%, 6%, 13% and 7% lower risk of ASCVD, respectively.

## Discussion

The present study investigated the association between LE8 and the risk of ASCVD and VTE among 1899 Finnish middle-aged and older men. The findings from this study suggest that higher LE8 scores are associated with a lower risk of ASCVD but not VTE. The baseline characteristics of the study participants showed that a considerable proportion of the study population were smokers and had a family history of CHD. This emphasizes the importance of implementing efficient interventions that focus on smoking cessation and lifestyle modifications among individuals who have a family history of CHD. Such interventions have the potential to mitigate the risk of developing ASCVD [26]. We also observed that men who developed ASCVD and VTE were relatively older and of lower SES compared to those with no events. Although the average LE8 score for all participants was approximately 60% of the possible maximum LE8 score of 800, this was not optimal as it falls within the second quartile of LE8 (Q2). Therefore, it is necessary to improve the LE8 score within the population. Furthermore, the risk of ASCVD could be lowered by 17% with a 50-point increase in LE8 score among participants. Notably for LE8 components, is that a 10-unit increase in blood glucose score provided the highest protection from ASCVD. Interventions targeting optimal blood glucose levels and control could play an important role for ASCVD prevention in Finland.

This study is the first attempt to examine the association between AHA’s latest LE8 score and risk of VTE and likely to be the pioneer study to evaluate the association between LE8 and risk of ASCVD among a northern European population. Our findings on the risk of ASCVD are in conformity with a recent study from a combined Chinese cohort (China-PAR project and Kailuan cohort study) which reported that increasing LE8 scores, in quintiles, decreased the 10-year and lifetime risks of ASCVD.(17) The Chinese participants with the highest quintile had more than 60% lower risk of ASCVD [17], whereas in this present study, Finnish participants within the highest quartile of LE8 score had 58% lower risk of ASCVD. Although results from a recent UK Biobank study [27] were related to a composite outcome of major adverse cardiovascular events (ischaemic heart disease, myocardial infarction, stroke and heart failure), the observation of higher risk of events among individuals with lower LE8 scores aligns with our findings. Our findings are consistent with earlier studies that have evaluated the association between LS7 (ideal cardiovascular health) and risk of ASCVD [16,28].

The LE8 score was not associated with the risk of VTE in the current study. To our knowledge, no other studies have examined this specific relationship. However, prior studies have utilized the previous version of the AHA's LS7 to investigate this association [14,15,29]. For instance, in the REGARDS study (Reasons for Geographic and Racial Differences in Stroke) [14], participants with optimal LS7 scores had lower risk of VTE. Similarly, the Multi-Ethnic Study of Atherosclerosis (MESA) found reduced risk of deep vein thrombosis or pulmonary embolism in those with an optimal LS7 score [15]. Despite VTE being the third leading vascular disease following myocardial infarction and stroke [4], the proportions were low (<10%) across different study cohorts that evaluated AHA’s cardiovascular metrics and risk of VTE – <1% for REGARDS [14], 3.3% for MESA [15], 5.2% for Atherosclerosis Risk in Communities Study (ARIC) [29] and 6.7% for KIHD (present study). Although VTE risk is reportedly more common in men than women [4], it is recommended to implement preventive measures aimed at reducing the risk of VTE in specific populations.

We further evaluated how LE8 factors independently associate with the risk of ASCVD and VTE. Apart from the diet and sleep health scores, we observed an indirect significant relation between a 10-unit rise in the other LE8 factor scores and the risk of ASCVD among KIHD study participants in the fully adjusted model. This conforms with Rana and colleagues’ work on LS7 and risk of ASCVD [28], which did not include diet and sleep health. Therefore, improving individual LE8 factor scores by at least 10 points may lower the risk of ASCVD in the population. Collectively, as a composite measure, LE8 score (50-unit increase) produced more protection (17%) from ASCVD than individual factors. This is not surprising, considering that the components of LE8 have significant associations with risk of ASCVD [30–32]. For example, moderate- and high- compared to low PA reduces the risk of ASCVD [32] through a multifactorial approach [33]; and the North Karelia Project in Finland, which implemented dietary changes to limit fat and salt intake contributed to the decrease in CVD mortality [34]. High BP and abnormal blood glucose levels are known risk factors for ASCVD, as they can cause damage to the blood vessels and increase the risk of plaque formation [35,36]. Within our study population, the mean score for diet was found to be the lowest, while the mean score for blood glucose was the highest. This suggests that improving the population’s diet score may require more extensive efforts, awareness and public health interventions compared to those needed for blood glucose. Therefore, our study findings provide a simplified framework for prioritizing targeted preventative interventions or programs for modifiable risk factors of ASCVD in the general population of Finland.

Findings of this study have important implications for clinical practice. Life’s Essential 8 provides a simple and effective tool for assessing individual’s cardio­vascular health and promoting healthy lifestyle behaviours. The LE8 metrics would not only benefit individuals to specifically identify modifiable risk factors but would highlight LE8 factors that can be directly changed for better and improved LE8 scores and better cardiovascular health. Healthcare providers can use this tool to educate patients on the importance of maintaining a healthy lifestyle and managing risk factors such as BP, blood glucose and PA levels. Furthermore, the individual components of LE8 can serve as targets for intervention and treatment to reduce the risk of ASCVD. However, as the study did not find a significant association between LE8 and VTE risk, this indicates that the LE8 metrics may not be as relevant in the context of VTE prevention. Therefore, we recommend that individuals and the general population should focus on improving the overall LE8 score rather than emphasizing only one component.

The uniqueness of being the first prospective study to assess the association of the newly updated LE8 and the risk of VTE adds strength to our study. Other strengths lie in its relatively large homogeneous sample size and the long follow-up period which is adequate for the assessment of the outcomes. The possibility of residual confounding and lack of data on VTE subtypes are limitations. Other limitations include the inability to generalize our research findings to other population groups, such as in women and other ethnicities; the risk of misclassification bias may be present due to the utilization of self-administered questionnaires for data collection on certain components of the LE8 and the use of baseline measures given that there could be changes over the period of follow-up due to ageing, comorbidities and lifestyle modification. The use of baseline data could underestimate the true associations due to regression dilution bias. Therefore, it would be interesting for future studies to investigate the longitudinal evolution of LE8 factors and explore interventions that improve lifestyle behaviours to improve LE8 scores and lower risk of ASCVD and possibly VTE.

## Conclusion

The findings of this study suggest that higher LE8 scores are associated with a lower risk of ASCVD but not VTE among men. The results highlight the importance of adopting healthy lifestyle behaviours and targeting multiple health factors to reduce the risk of ASCVD. These findings suggest that the effects of a combined algorithm of lifestyle behaviours and health factors on cardiovascular risk may be complex and may depend on the specific outcome being studied. Whether a population-wide approach of LE8 to reduce CVD risk and its associated burden will have impact on VTE risk reduction requires further evaluation.

## Supplementary Material

Supplemental Material

## Data Availability

The data underlying this article were provided by University of Eastern Finland, Institute of Public Health and Clinical Nutrition by permission. Data will be shared upon request to the corresponding author with permission of University of Eastern Finland.
