# Supplementary material for: Life’s Essential 8 is associated with atherosclerotic cardiovascular disease but not venous thromboembolism in men: a prospective cohort study
Source: Ann Med. 2023 Jul 17;55(1):2233894. doi: 10.1080/07853890.2023.2233894 (PMC10353322; doi:10.1080/07853890.2023.2233894)
Supplement: Supplemental Material [file IANN_A_2233894_SM1057.pdf]

Supplement Table 1. The American Heart Association's Life's Essential 8

Domain	CVH metric	Quantification of CVH metric	
Health behaviours	Diet	Points	MEPA score
		100	15-16
		80	12-14
		50	8-11
		25	4-7
		0	0-3
	Physical Activity	Points	Minutes
		100	≥150
		90	120-149
		80	90-119
		60	60-89
		40	30-59
		20	1-29
		0	0
	Nicotine Exposure	Points	Status
		100	- Never smoked
		75	- Former smoker, quit ≥5years
		50	- Former smoker, quit 1 to < 5years
		25	- Former smoker, quit <1 year, or currently using inhaled NDS
		0	- Current smoker
	Sleep Health	Points	Level (Average hours of sleep per night)
		100	7 to <9
		90	9 to <10
		70	6 to <7
		40	5 to <6 or ≥10
		20	4 to <5
		0	<4
Health Factors	Body mass index	Points	Level (kg/m^2)
		100	<25
		70	25.0-29.9
		30	30.0-34.9
		15	35.0-39.9
		0	≥40.0
	Blood lipids	Points	Level (Non-HDLc, mg/dL)
		100	<130
		60	130-159
		40	160-189
		20	190-219
		0	≥220
		Subtract 20 points	If drug-treated level
	Blood glucose	Points	Level (FBG, mg/dL or HbA1c, %)
		100	- No history of diabetes and FBG <100 (or HbA1c <5.7)
		60	- No diabetes and FBG 100-125 (or HbA1c 5.7-6.4) (prediabetes)
		40	- Diabetes with HbA1c <7.0
		30	- Diabetes with HbA1c 7.0-7.9
		20	- Diabetes with HbA1c 8.0-8.9
		10	- Diabetes with HbA1c 9.0-9.9
		0	- Diabetes with HbA1c ≥10
	Blood pressure	Points	Level (SBP and/or DBP, mmHg)
		100	• <120/<80(optimal)
		75	• 120-129/<80 (elevated)
		50	• 130-139 or 80-89 (stage 1 hypertension)
		25	• 140-159 or 90-99
		0	• ≥160 or ≥100
		Subtract 20 points	If treated level

NDS, nicotine delivery systems

For Peer Review Only

Supplement Table 2: The Mediterranean Eating Pattern for Americans (MEPA) score in KIHD Study

Mediterranean-like diet factor	Measure	Score
≥2 servings of olive oil per day	1 serving = 1 tablespoon = 14 g	1
≥7 servings of green leafy vegetables per week	1 serving = 75 g	1
≥2 servings of other vegetables per day	1 serving = 75 g	1
≥2 servings of berries per week	1 serving = 150 g	1
≥1serving of other fruit per day	1 serving = 150 g	1
≤3 servings of red meat, hamburger, bacon, or sausage per week	1 serving = 65 g	1
≥1serving of fish per week	1 serving = 100 g	1
≤5 servings of chicken per week	1 serving = 80 g	1
≤4 servings of full fat or regular cheese or cream cheese per week	1 serving = 40 g	1
≤5 servings of butter or cream per week	1 serving = 20 g	1
≥3 servings of beans per week	1 serving = 75 g.	1
≥3servings of whole grains per day	1 serving = 40 g (bread), 100 g (rice and pasta), 120 g (porridge).	1
≤4 servings of commercial sweets, candy bars, pastries, cookies, or cakes per week	1 serving = 30g.	1
≥4 servings of nuts per week	1 serving = 30 g	1
≤1meal at a fast-food restaurant per week		1
>0 or ≤2 servings of alcohol per day for men and >0 or ≤1 serving of alcohol per day for women	1 serving = 14 grams of pure alcohol.	1
**Total MEPA Score**		**16**

For Peer Review Only

Supplement Table 3. LE8 association with risk of ASCVD and VTE excluding participants with CVD history

	**ASCVD**			**VTE**		
	n/N	Model 1	Model 2	n/N	Model 1	Model 2
	730/1611	HR(95%CI); *P* value	HR(95%CI); *P* value	107/1611	HR(95%CI); *P* value	HR(95%CI); *P* value
**LE8 (in quartiles, Q)**						
Q1	220/410	1(Ref.)	1(Ref.)	21/410	1(Ref.)	1(Ref.)
Q2	199/426	0.71 (0.58-0.85); <0.01	0.74 (0.61-0.89); <0.01	25/426	0.98 (0.55-1.74); 0.93	0.99 (0.55-1.77); 0.97
Q3	171/383	0.55 (0.46-0.68); <0.01	0.59 (0.48-0.72); <0.01	35/383	1.48 (0.86-2.54); 0.16	1.47 (0.85-2.54); 0.17
Q4	140/392	0.41 (0.33-0.51); <0.01	0.45 (0.36-0.56); <0.01	26/392	1.01 (0.57-1.80); 0.97	0.99 (0.55-1.78); 0.97
**Total LE8 score (per 50-unit increase)**	730/1611	0.82 (0.80-0.86); <0.01	0.84 (0.81-0.88); <0.01	107/1611	1.02(0.92-1.14); 0.68	1.02 (0.91-1.14); 0.75

ASCVD, atherosclerotic cardiovascular disease; CI, Confidence interval; HR, Hazard ratio; KIHD, Kuopio Ischaemic Heart Disease; LE8, Life's Essential 8; n/N, number of events/Total; VTE, Venous thromboembolism
Model 1, Adjusted for age
Model 2, Model 1 plus alcohol consumption, socioeconomic status, family history of coronary heart disease and history of cancer
LE8 Scores: Quartile 1, ≤ 420; Quartile 2, >420 to 485; Quartile 3, >485 to 550; Quartile 4, >550

For Peer Review Only
